# Visualization and quantitation of GLUT4 translocation in human skeletal muscle following glucose ingestion and exercise

**DOI:** 10.14814/phy2.12375

**Published:** 2015-05-11

**Authors:** Helen Bradley, Christopher S Shaw, Claus Bendtsen, Philip L Worthington, Oliver J Wilson, Juliette A Strauss, Gareth A Wallis, Alice M Turner, Anton JM Wagenmakers

**Affiliations:** 1 School of Sport, Exercise and Rehabilitation Sciences, University of BirminghamBirmingham, UK; 2 School of Exercise and Nutrition Sciences, Deakin UniversityGeelong, Vic., Australia; 3 Computational Biology, Discovery Sciences, AstraZeneca R&DCambridge, UK; 4 Computational Biology, Discovery Sciences, AstraZeneca R&DAlderley Park, Macclesfield, UK; 5 Institute for Sport, Physical Activity and Leisure, Leeds Beckett UniversityLeeds, UK; 6 Research Institute for Sport and Exercise Sciences, Liverpool John Moores UniversityLiverpool, UK; 7 School of Clinical and Experimental Medicine, University of BirminghamBirmingham, UK; 8 Heart of England NHS Foundation TrustBordesley Green East, Birmingham, UK

**Keywords:** Exercise, glucose ingestion, GLUT4 translocation, skeletal muscle

## Abstract

Insulin- and contraction-stimulated increases in glucose uptake into skeletal muscle occur in part as a result of the translocation of glucose transporter 4 (GLUT4) from intracellular stores to the plasma membrane (PM). This study aimed to use immunofluorescence microscopy in human skeletal muscle to quantify GLUT4 redistribution from intracellular stores to the PM in response to glucose feeding and exercise. Percutaneous muscle biopsy samples were taken from the *m. vastus lateralis* of ten insulin-sensitive men in the basal state and following 30 min of cycling exercise (65% VO_2 max_). Muscle biopsy samples were also taken from a second cohort of ten age-, BMI- and VO_2 max_-matched insulin-sensitive men in the basal state and 30 and 60 min following glucose feeding (75 g glucose). GLUT4 and dystrophin colocalization, measured using the Pearson's correlation coefficient, was increased following 30 min of cycling exercise (baseline *r *=* *0.47 ± 0.01; post exercise *r *=* *0.58 ± 0.02; *P* < 0.001) and 30 min after glucose ingestion (baseline *r *=* *0.42 ± 0.02; 30 min *r *=* *0.46 ± 0.02; *P* < 0.05). Large and small GLUT4 clusters were partially depleted following 30 min cycling exercise, but not 30 min after glucose feeding. This study has, for the first time, used immunofluorescence microscopy in human skeletal muscle to quantify increases in GLUT4 and dystrophin colocalization and depletion of GLUT4 from large and smaller clusters as evidence of net GLUT4 translocation to the PM.

## Introduction

Meal-induced increases in plasma insulin and muscle contractions during moderate intensity exercise are potent physiological stimulators of plasma glucose uptake into skeletal muscle (Katz et al. 1983; Ferrannini et al. 1985; van Loon et al. 2001; Wasserman et al. 2011). Leg glucose uptake increased threefold during the 4 h period following oral ingestion of 92 g of glucose (Katz et al. 1983), appr-oximately fivefold during a hyperinsulinemic-euglycemic clamp (DeFronzo et al. 1985) and approximately 15-fold during exercise at 50–60% VO_2 max_ (Katz et al. 1986; Martin et al. 1995).

Glucose is transported into the muscle cell via facilitated transport through glucose transporter proteins in the plasma membrane (PM) and T-tubule membranes (Bell et al. 1993). Glucose transporter 4 (GLUT4) is an insulin and contraction responsive glucose transporter and is the major glucose transporter isoform expressed in skeletal muscle (Mueckler 1994; Gaster et al. 2000). Global or muscle-specific GLUT4 knockout in mice resulted in reduced basal muscle glucose uptake and reduced insulin and contraction-stimulated muscle glucose uptake in vitro, demonstrating the vital role of GLUT4 in blood glucose homeostasis (Ryder et al. 1999a,b; Zisman et al. 2000).

Previous studies investigating GLUT4 translocation in human skeletal muscle in response to increases in plasma insulin concentrations or moderate intensity exercise have used density gradient centrifugation methods with the aim to isolate pure PM fractions and measure their GLUT4 content with western blots. In insulin-sensitive individuals, these studies have demonstrated increases in GLUT4 PM content of 27% 1 h following ingestion of 75 g of glucose (Goodyear et al. 1996) and of 60% 30–40 min after the start of a hyperinsulinemic-euglycemic clamp (Guma et al. 1995). In another study 45–60 min of exercise at 60–70% VO_2 max_ resulted in a 71% increase in GLUT4 content in the PM fraction (Kennedy et al. 1999).

A series of detailed immunofluorescence and electron microscopy studies in rat and mouse skeletal muscle suggest that GLUT4 is present predominantly at the fiber periphery and in perinuclear regions in the basal state. GLUT4 appeared as large and small storage clusters which are likely pretethered at the PM and T-tubule membranes (Ploug et al. 1998; Lauritzen et al. 2006, 2008; Lizunov et al. 2012). GLUT4 storage depots have been characterized as being at the membranes of the trans-Golgi network, in endosomal membranes and in GLUT4 storage vesicles (GSVs) (Rodnick et al. 1992; Ploug et al. 1998; Lizunov et al. 2012). Furthermore, studies in transgenic mouse muscle fibers have used labeling of exofacially tagged GLUT4 to demonstrate a small amount of PM GLUT4 in the basal state, which increases 2–4-fold following insulin stimulation or contraction (Schertzer et al. 2009; Lizunov et al. 2012). A redistribution of GLUT4 from intracellular clusters to the PM and T-tubule membranes has also been reported following insulin injection and electrical muscle stimulation in in vivo studies of rodent muscle transiently transfected with GFP-tagged GLUT4 (Ploug et al. 1998; Lauritzen et al. 2006, 2010).

Both increases in plasma insulin and moderate intensity exercise recruit GLUT4 from large and small clusters (Ploug et al. 1998). During the first 30 min of insulin stimulation large (>1 *μ*m) GLUT4 clusters did not move *in toto*, rather they were stationary and locally depleted of GLUT4, which probably reflects budding off of microvesicles as observed by Lizunov et al. (2012) in mouse muscle. Furthermore, following insulin stimulation in isolated mouse muscles that endogenously expressed HA-GLUT4-GFP, the majority of insulin-stimulated GLUT4 vesicle fusion events emanated from pretethered vesicles, while the small pool of mobile GLUT4 vesicles was not affected by insulin (Lizunov et al. 2012).

We have recently adapted existing confocal immunofluorescence microscopy methods for visualization of GLUT4 in human skeletal muscle to negate the methodological flaws inherent in membrane fractionation studies by measuring the colocalization of GLUT4 with the PM marker dystrophin (Bradley et al. 2014). In this study we also showed that 6 weeks of endurance training and high-intensity interval training led to similar size increases in GLUT4 clusters specifically in the PM layer and 1 *μ*m layer immediately adjacent to the PM (Bradley et al. 2014), which is the site from which most of the PM-GLUT4 fusion events emanate in animal studies (Lizunov et al. 2012). The aim of the current study was to visualize and characterize the translocation of GLUT4 in human skeletal muscle using this method in response to physiological stimuli known to elevate rates of muscle glucose uptake. We specifically investigated changes in the colocalization of GLUT4 with the PM marker dystrophin and changes in the distribution and size of large and small GLUT4 stores that occur in response to 30 min of moderate intensity exercise and 30 and 60 min following glucose ingestion. We tested the hypotheses that GLUT4 and dystrophin colocalization would increase in response to both glucose ingestion and moderate intensity exercise and that the large and small GLUT4 stores would decrease in abundance and size, particularly in the 1 *μ*m layer immediately adjacent to the PM.

## Materials and Methods

The data presented in this paper were obtained from two separate participant cohorts and sets of experiments, which are termed experiment 1 and experiment 2.

### Ethics, recruitment, and informed consent

Ethical approval was granted by Birmingham East, North, and Solihull and West Midlands Black Country NHS Research Ethics Committees. Ten healthy, lean, recreationally active participants were recruited for each experiment (mean ± SEM, experiment 1 age 21 ± 1 year, BMI 23.1 ± 0.4 kg m^−2^, VO_2 max_ 52.5 ± 2.4 mL min^−1^ kg^−1^, experiment 2 age 25 ± 2 years, BMI 22.6 ± 0.5 kg m^−2^, VO_2 max_ 50.7 ± 2.2 mL min^−1^ kg^−1^) and written informed consent was obtained prior to their commencement in the study.

### Study protocol

All participants performed an incremental exercise test to exhaustion on an electronically braked cycle ergometer (Lode BV, Groningen, The Netherlands) to determine their maximal aerobic capacity (VO_2 max_) using an online gas collection system (Oxycon Pro, Jaeger, Germany). Workload began at 95 W and increased by 35 W every 3 min until volitional fatigue.

### Experiment 1

Participants completed two visits and were asked to refrain from strenuous physical activity for 48 h prior to arrival at the laboratory at 7 am following an overnight fast in both cases. During the first visit participants completed an oral glucose tolerance test (OGTT) to measure insulin sensitivity using the ISI-Matsuda (Matsuda and DeFronzo 1999). A blood sample was drawn from a forearm vein and participants ingested 75 g glucose as a 25% solution. Subsequent blood samples were drawn 30, 60, 90, and 120 min after glucose ingestion. During the second visit (at least 1 week after the first) a blood sample was drawn from a forearm vein and a percutaneous needle biopsy sample (Bergstrom 1975) was taken from the *m. vastus lateralis* in the basal state under local anesthesia (1% lidocaine). To investigate contraction-mediated GLUT4 translocation participants completed 30 min cycling on an electronically braked cycle ergometer at a workload equivalent to 65% VO_2 max_ and a second needle biopsy was taken immediately upon completion of exercise. Blood samples were taken after 15 and 30 min of exercise.

### Experiment 2

Participants were again asked to refrain from strenuous physical activity for 48 h prior to the visit and arrived at the laboratory at 7 am following an overnight fast. A percutaneous needle biopsy sample was taken from the *m. vastus lateralis* in the basal state under local anesthesia (1% lidocaine) and a blood sample was drawn from a forearm vein. Participants then ingested 75 g glucose as a 25% solution and subsequent needle biopsy samples were taken from the *m. vastus lateralis* 30 and 60 min after glucose ingestion. Blood samples were drawn 30, 60, 90, and 120 min after glucose ingestion.

### Sample collection and storage

Immediately after collection, muscle biopsy samples were blotted to remove excess blood and any visible collagen or fat was discarded. The sample was embedded in Tissue Tek OCT (Sakura, USA) compound and immediately frozen in liquid nitrogen-cooled isopentane (Sigma Aldrich, UK). The sample was then transferred to an aluminum cryotube (Caltag Medsystems, UK) for storage at −80°C. Blood samples were collected into ethylenediaminetetraacetic acid (EDTA)-containing tubes (BD vacutainer, USA) and kept on ice before centrifuging for 15 min at 3500 rpm at 4°C. Plasma was aliquoted and stored at −80°C.

### Plasma glucose and insulin analysis

Plasma glucose was analyzed in duplicate in all samples spectrophotometrically using an ILab 600 analyzer and the glucose oxidase kit (Instrumentation Laboratory Ltd, UK). Plasma insulin was analyzed in duplicate in all samples using commercially available insulin ELISA kits (Invitrogen, UK).

### Immunofluorescence staining and microscopy

The full protocol and GLUT4 antibody validation was described previously (Bradley et al. 2014) and therefore is outlined in brief below. Frozen muscle biopsy samples were cryosectioned (Bright Instrument Company Limited, Huntingdon, UK) to a thickness of 5 *μ*m onto uncoated glass microscope slides (VWR international, Leicester, UK). For each experiment, three slides were processed for GLUT4 and dystrophin. All sections were fixed in 75% acetone and 25% ethanol solution for 5 min and were subsequently washed 3 times for 5 min in phosphate-buffered saline (PBS, 137 mmol L^−1^ sodium chloride, 3 mmol L^−1^ potassium chloride, 8 mmol L^−1^ sodium phosphate dibasic, 3 mmol L^−1^ potassium phosphate monobasic). GLUT4 primary antibody (Abcam, Cambridge, UK) was applied to sections at a 1 in 200 dilution in combination with a dystrophin primary antibody (Sigma Aldrich, St Louis, MO) at a 1 in 400 dilution for 2 h at room temperature. Following primary antibody incubation, sections were washed 3 times for 5 min in PBS and were then incubated in 1 in 200 dilutions of secondary antibodies for 30 min at room temperature. AlexaFluor 488-conjugated goat anti-rabbit IgG secondary antibody (Invitrogen, UK) was used to detect GLUT4 primary antibody while AlexaFluor 594-conjugated goat anti-mouse IgG2b (Invitrogen, Paisley, UK) was used to detect dystrophin primary antibody. Sections were then washed 3 times for 5 min in PBS and glass coverslips were mounted with 20 *μ*L mowiol mounting medium [6 g glycerol (Sigma Aldrich), 2.4 g mowiol 4–88 (Sigma Aldrich) and 0.026 g 1,4-Diazabicyclo[2.2.2]octane (DABCO) (Sigma Aldrich) dissolved in 18 mL 0.2 M Tris-buffer (pH 8.5) (Sigma Aldrich)]. Images were captured using an inverted confocal microscope (Leica DMIRE2; Leica Microsystems, Wetzlar, Germany) with a 63× oil immersion objective (1.4 NA). AlexaFluor 488 fluorophores were excited with a 488 nm line of the argon laser for excitation and 498–571 nm emission. AlexaFluor 594 fluorophores were excited with the 594 nm line of the helium–neon laser for excitation and 601–713 nm emission.

### Immunofluorescence image analysis

For analysis of GLUT4 and dystrophin colocalization, images were processed and analyzed using Image-Pro Plus 5.1 software (Media Cybernetics, Rockville, MD), as described previously (Bradley et al. 2014). All image processing and analysis was kept consistent between images within each experiment. An average of 51 ± 2 muscle fibers was analyzed for each participant. In order to analyze PM GLUT4 the Pearson's correlation coefficient between each pair of GLUT4 and dystrophin images were calculated using the colocalization tool in Image-Pro Plus 5.1. The Pearson's correlation coefficient is a measure of the correlation of the intensity of green (GLUT4) and red (dystrophin) signal in each pixel of each image and is an accepted and commonly used method to quantify the degree of colocalization of two markers using immunofluorescence microscopy (Dunn et al. 2011).

Quantitation of GLUT4 in the PM layer (dystrophin-stained region) and five 1 *μ*m layers in from the PM was carried out in MATLAB (v. 2012b, The MathWorks Inc., Natick, MA, 2012) using a bespoke image analysis algorithm, as described previously (Bradley et al. 2014). The fibers were segmented in the dystrophin image using the active contour, or snake, approach (Kass et al. 1988) and a distance map from the contour was used to generate a 2.5 pixel thick region to cover the dystrophin-stained region. This region has been designated the PM layer. Subsequently five 1 *μ*m thick layers were generated inside the fiber. GLUT4 large and small spots were identified using intensity and size thresholds within each region (large >1 *μ*m, small <1 *μ*m diameter).

### Statistical analysis

Plasma glucose and insulin data were analyzed for statistical significance using a repeated measures anova with post hoc Bonferroni pairwise comparisons. Plasma glucose and insulin data were compared between experiment 1 and 2 participants using an independent *t* test. The Pearson's correlation coefficient data from immunofluorescence images for experiment 1 were analyzed for statistical significance using a paired *t* test. For experiment 2 a repeated measures ANOVA was used to compare the Pearson's correlation coefficient at baseline and 30 min and 60 min after glucose ingestion, with post hoc Bonferroni pairwise comparisons. A repeated measures ANOVA was used to investigate the effect of stimulation and cell layer on number and size of large and size of small GLUT4 spots. In the case of significant main effects, post hoc Bonferroni pairwise comparisons were made and where significant were marked on the graph. In the case of significant interactions, which occurred only in the experiment 2 data, repeated measures ANOVAs were carried out within each layer and significant Bonferroni pairwise comparisons were marked on the graph. The subject characteristics of the two groups were compared using an independent *t* test. Data are displayed as mean ± SE.

## Results

### Subject characteristics

Subject characteristics are shown in Table1. Ten healthy, lean, recreationally active subjects were recruited separately for experiments 1 and 2. All subjects were insulin sensitive, according to classification of fasting plasma glucose of less than 5.6 mmol L^−1^ and 2 h OGTT plasma glucose of less than 7.8 mmol L^−1^ (Genuth et al. 2003). There were no significant differences in VO_2 max_, BMI, fasting plasma glucose, fasting plasma insulin or ISI-Matsuda between the cohorts for experiments 1 and 2.

**Table 1:** Subject characteristics

	Experiment 1 (*N* = 10)	Experiment 2 (*N* = 10)
Age (year)	21 ± 1	25 ± 2
Height (m)	1.81 ± 0.02	1.83 ± 0.01
Body mass (kg)	75.9 ± 2.4	75.3 ± 2.0
BMI (kg m^−2^)	23.1 ± 0.4	22.6 ± 0.5
VO_2 max_ (mL min^−1^ kg^−1^)	52.5 ± 2.4	50.7 ± 2.2
W_max_ (W)	278 ± 15	285 ± 13
Fasting plasma glucose (mmol L^−1^)	5.3 ± 0.1	5.1 ± 0.2
2 h OGTT plasma glucose (mmol L^−1^)	5.0 ± 0.3	5.4 ± 0.4
Fasting plasma insulin (*μ*IU mL^−1^)	14.3 ± 1.4	12.7 ± 1.7
2 h OGTT plasma insulin (*μ*IU mL^−1^)	31.4 ± 4.2	41.4 ± 7.3#
ISI-Matsuda	3.1 ± 0.3	4.4 ± 0.6

Mean ± SE, ^#^Significant difference to fasting insulin concentration, *P* < 0.05, repeated measures ANOVA.

### Basal state GLUT4 localization

**Figure 1:**
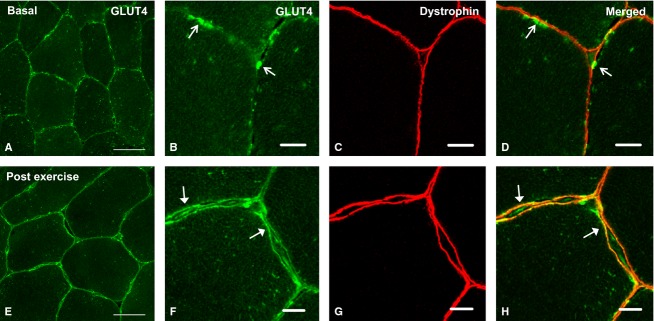
Representative confocal GLUT4 immunofluorescence images of human skeletal muscle fibers in the basal state (A–D) and postexercise (E–H) from experiment 1. Images A and E show GLUT4 localization in green (scale bars 50 *μ*m). Images B and F show more detailed images of GLUT4 in PM regions (scale bars 10 *μ*m). Images C and G show the PM marker dystrophin in red (scale bars 10 *μ*m). Merged images in D and H demonstrate colocalization of GLUT4 with the PM marker dystrophin (scale bars 10 *μ*m). Open arrows indicate clusters of GLUT4 at the PM, while filled arrow heads indicate GLUT4 localized to and equally dispersed in the PM.

Consistent with previous rodent studies (Rodnick et al. 1992; Ploug et al. 1998; Lauritzen et al. 2006, 2008) and as shown in our recent publication (Bradley et al. 2014), GLUT4 immunofluorescence staining (Figs.1A,B and 4A, B) is observed in large clusters, as well as smaller punctuate structures which at a lower magnification appear as a diffuse background stain. The large clusters appear at the fiber periphery as well as in intracellular regions. Immunofluorescence staining of the protein dystrophin (Figs.1C and 4C) was used to mark the PM. Figs.1D and 4D show the colocalization of GLUT4 and dystrophin in the basal state.

### Experiment 1: GLUT4 localization in response to 30 min moderate intensity exercise

There were no significant changes in plasma glucose (baseline 5.4 ± 0.1 mmol L^−1^, 15 min 5.0 ± 0.1 mmol L^−1^, 30 min 5.2 ± 0.2 mmol L^−1^, *P > *0.05) or insulin concentrations (baseline 14.5 ± 2.2 *μ*IU mL^−1^, 15 min 13.9 ±2.4 *μ*IU mL^−1^, 30 min 11.0 ± 2.0 *μ*IU mL^−1^, *P > *0.05) in response to the exercise bout.

**Figure 2:**
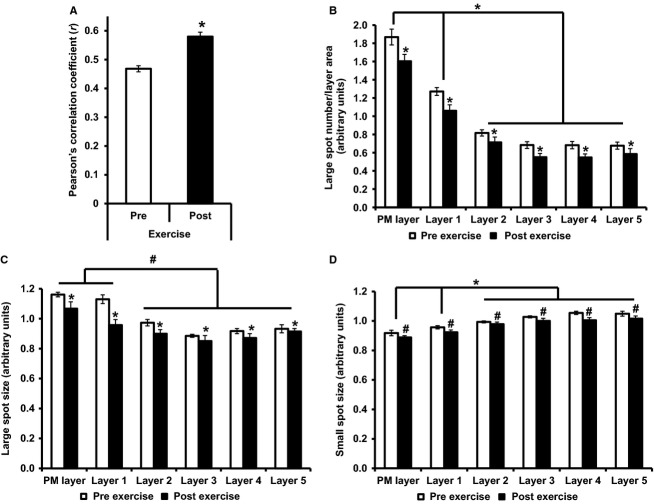
GLUT4 localization pre- and post-30 min exercise at 65% VO_2 max_. (A) Colocalization of GLUT4 with PM marker dystrophin, measured using the Pearson's correlation coefficient, paired *t* test *P < 0.001. (B) Large GLUT4 spot number, repeated measures ANOVA stimulation effect *P = *0.005, cell layer effect *P < *0.001, interaction *P = *0.065, post hoc Bonferroni *P < 0.01, ^#^*P < *0.05. (C) Large GLUT4 spot size, repeated measures ANOVA stimulation effect *P = *0.001, cell layer effect *P = *0.001, interaction *P = *0.131, post hoc Bonferroni *P < 0.01, ^#^*P < *0.05. (D) Small GLUT4 spot size, repeated measures ANOVA stimulation effect *P = *0.038, cell layer effect *P < *0.001, interaction *P = *0.419 post hoc Bonferroni *P < 0.01, ^#^*P < *0.05. Graphs show mean ± SE,* N* = 10.

Following 30 min of cycling exercise at 65% of VO_2 max_ the GLUT4 immunofluorescence signal demonstrated a clear visual redistribution to the PM (Fig.1E,F). Postexercise, GLUT4 (Fig.1H) exhibited a continuous and homogeneous colocalization with the PM marker dystrophin, while in the resting sample (Fig.1D) GLUT4 and dystrophin colocalization at the PM was present only in clusters. The Pearson's correlation coefficient increased significantly from *r *=* *0.47 ± 0.01 before exercise to *r *=* *0.58 ± 0.02 after exercise (*P < *0.001), indicating an increase in GLUT4 and dystrophin colocalization and therefore a redistribution of GLUT4 to the PM following 30 min exercise (Fig.2). Large GLUT4 spot number (stimulation effect *P = *0.005, cell layer effect *P < *0.001, interaction *P = *0.065) and spot size (stimulation effect *P = *0.001, cell layer effect *P = *0.001, interaction *P = *0.131) and small GLUT4 spot size (stimulation effect *P = *0.038, cell layer effect *P < *0.001, interaction *P = *0.419) in the PM layer and five 1 *μ*m intracellular layers was reduced following exercise.

### Experiment 2: GLUT4 localization in response to glucose ingestion

**Figure 3:**
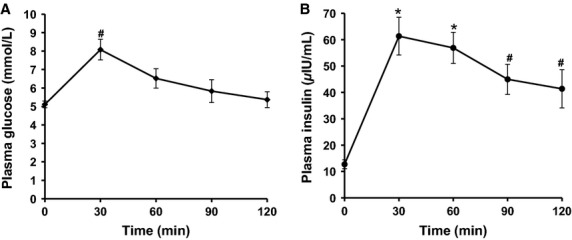
Plasma glucose and insulin during OGTT for experiment 2. Data presented are mean ± SE. *N* = 10. Repeated measures ANOVA glucose *P < *0.001 (A), insulin *P < *0.001 (B), post hoc Bonferroni pairwise comparisons *P < 0.01, ^#^*P < *0.05.

Figure3 displays plasma glucose and insulin concentrations during OGTT in experiment 2. Plasma glucose incre-ased above baseline at 30 min (baseline 5.1 ± 0.2 mmolL^−1^, 30 min 8.1 ± 0.6 mmol L^−1^, *P < *0.05) and returned to baseline by 60 min (6.5 ± 0.5 mmol L^−1^, *P > *0.05). Plasma insulin peaked at 30 min increasing above baseline (baseline 12.7 ± 1.7 *μ*IU mL^−1^, 30 min 61.3 ± 7.2 *μ*IUmL^−1^, *P < *0.001) and remained increased above baseline at 120 min (41.4 ± 7.3 *μ*IU mL^−1^, *P < *0.05).

**Figure 4:**
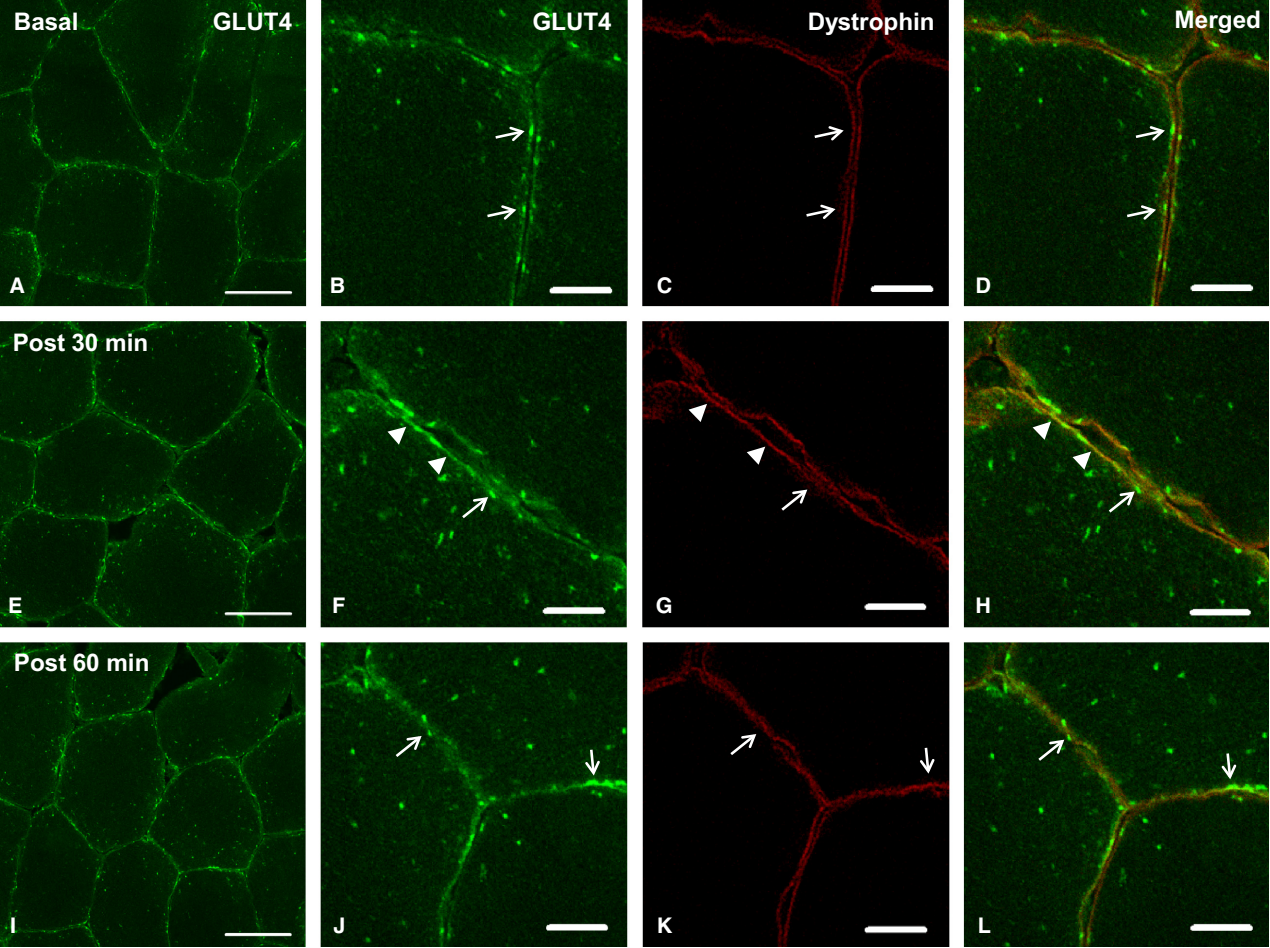
Representative confocal immunofluorescence images of GLUT4 immunofluorescence in human skeletal muscle fibers in the basal state (A, B), 30 min post glucose ingestion (E, F) and 60 min postglucose ingestion (I, J) from experiment 2. Images A, E, and I show GLUT4 localization in green (scale bars 50 *μ*m). Images B, F, and J show more detailed images of GLUT4 in PM regions (scale bars 10 *μ*m). Images C,G, and K show the PM marker dystrophin in red (scale bars 10 *μ*m). Merged images in D, H, and L demonstrate colocalization of GLUT4 with the plasma membrane marker dystrophin (scale bars 10 *μ*m). Open arrows indicate clusters of GLUT4 at the PM, while filled arrow heads indicate moderate continuous GLUT4 signal at the PM.

**Figure 5:**
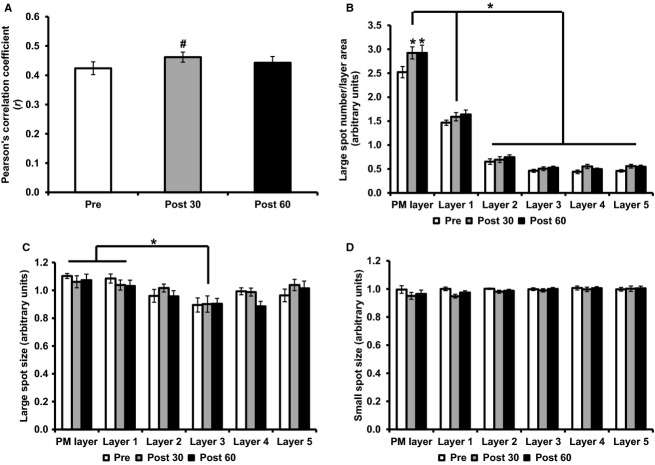
GLUT4 localization pre and 30 min and 60 min postglucose ingestion. (A) Colocalization of GLUT4 with PM marker dystrophin, measured using the Pearson's correlation coefficient. Repeated measures ANOVA *P = *0.008, *post hoc Bonferroni pairwise comparisons *P < *0.05. (B) Large GLUT4 spot number, repeated measures ANOVA stimulation effect *P = *0.001, cell layer effect *P < *0.001, interaction *P = *0.012, post hoc Bonferroni *P < 0.01, ^#^*P < *0.05. (C) Large GLUT4 spot size, repeated measures ANOVA stimulation effect *P = *0.487, cell layer effect *P < *0.001, interaction *P = *0.588, post hoc Bonferroni ^#^*P* < 0.05. (D) Small GLUT4 spot size, repeated measures ANOVA stimulation effect *P = *0.055, cell layer effect *P = *0.404, interaction *P = *0.086. Graphs show mean ± SE,* N* = 10.

To investigate the time course of GLUT4 translocation in response to glucose ingestion, GLUT4 and dystrophin colocalization was investigated in the basal state and 30 and 60 min after glucose ingestion (Fig.4). Thirty minutes after glucose ingestion, GLUT4 clusters localized to the PM remained clearly visible with the additional appearance of moderate continuous staining along the PM (Fig.4E–H). The Pearson's correlation coefficient values to measure GLUT4 and dystrophin colocalization increased significantly from baseline at 30 min postglucose ingestion, while a nonsignificant increase was seen at 60 min (pre *r *=* *0.42 ± 0.02, 30 min *r *=* *0.46 ± 0.02, 60 min *r *=* *0.44 ± 0.02, repeated measures ANOVA *P = 0.008*, Fig.5), therefore indicating a redistribution of GLUT4 to the PM 30 min after glucose ingestion. Large GLUT4 spot number increased in the PM layer at 30 and 60 min following glucose ingestion (stimulation effect *P = *0.001, cell layer effect *P < *0.001, interaction *P = *0.012), while large GLUT4 spot size (stimulation effect *P = *0.487, cell layer effect *P < *0.001, interaction *P = *0.588) and small GLUT4 spot size (stimulation effect *P = *0.055, cell layer effect *P = *0.404, interaction *P = *0.086) did not change following glucose ingestion.

## Discussion

### Immunofluorescence visualization of insulin and contraction-mediated GLUT4 translocation

We have recently used confocal immunofluorescence microscopy methods to visualize GLUT4 in human skeletal muscle (Bradley et al. 2014). Visualization of GLUT4 in human skeletal muscle negates the methodological flaws inherent in membrane fractionation studies and provides information about the distribution of GLUT4 in large and small clusters and the PM. In addition we use the PM marker dystrophin to assess colocalization of GLUT4 with a PM marker and quantify this colocalization using the Pearson's correlation coefficient. We interpret significant increases in Pearson's correlation coefficient following glucose feeding or exercise to indicate GLUT4 translocation to the sarcolemma has occurred. In the current study, we have used these methods to assess depletion of large and small GLUT4 stores and GLUT4 translocation to the PM in human skeletal muscle 30 min following glucose ingestion and after 30 min of moderate intensity exercise.

Exercise at 60–70% VO_2 max_ has been shown to increase PM GLUT4 by 71% in human skeletal muscle using cellular subfractionation methods (Kennedy et al. 1999). In addition two successive periods of 5 min of electrical stimulation of rodent muscle at a frequency and voltage which recruits all the muscle fibers induced a significant increase in PM GLUT4 content measured using fluorescence microscopy (Ploug et al. 1998; Lauritzen et al. 2010). In line with these previous observations, the immunofluorescence images presented in this study show marked redistribution of GLUT4 to PM regions in response to 30 min moderate intensity cycling exercise in human volunteers. This resulted in the clear appearance of a continuous and homogeneous GLUT4 signal along the muscle cell border following exercise, which colocalized with the PM marker dystrophin (Fig.1). As this colocalization pattern of GLUT4 and dystrophin was not seen in the preexercise biopsies, the appearance of the continuous and homogeneous distribution of GLUT4 at the PM following exercise likely represents the dispersal of GLUT4 molecules in the PM from the original fusion site of the GLUT4 clusters (Lizunov et al. 2012). The conclusion that GLUT4 fusion and dispersal in the PM occurred following exercise is supported by the significant increase in GLUT4 colocalization with the PM marker dystrophin measured using the Pearson's correlation coefficient (Fig.2) and also by the significant decreases in the number and size of large GLUT4 spots and size of small GLUT4 spots in the 1 *μ*m layers immediately adjacent to the PM.

The appearance of continuous GLUT4 staining on the muscle cell border was also observed 30 min following glucose ingestion (Fig.4) and was confirmed by a significant increase in GLUT4-dystrophin colocalization measured using the Pearson's correlation coefficient (Fig.5). This finding was expected given that skeletal muscle accounts for the majority of insulin-mediated glucose disposal (Katz et al. 1983; Ferrannini et al. 1985) and GLUT4 is the main insulin-responsive GLUT isoform in skeletal muscle (Mueckler 1994). These findings are in line with previous observations of increased PM GLUT4 content in human muscle 30 min following a euglycemic-hyperinsulinemic clamp (Guma et al. 1995) and the rapid induction of GLUT4 translocation in rodent muscle following insulin injection (Lauritzen et al. 2006). However, we did not observe a significant increase above baseline in GLUT4 colocalization with the PM marker dystrophin at 60 min following glucose ingestion. This finding is in contrast to previous studies using subcellular fractionation techniques, which reported a 27% increase in GLUT4 in the PM 60 min after ingestion of an OGTT load (Goodyear et al. 1996). As even the 27% increase observed by Goodyear et al. (1996) is only a fraction of the threefold increase in skeletal muscle glucose uptake (Katz et al. 1983) observed in humans 60 min following glucose ingestion, we suggest that a gradual increase in the recruitment of muscle capillaries contributes significantly to the elevated glucose uptake at 60 min. Recruitment of muscle capillaries is regarded as an established mechanism to increase the capillary surface area available for glucose transport and thus enhance glucose uptake in skeletal muscle fibers in rodents and humans (Keske et al. 2014; Wagenmakers et al. 2015).

Interestingly, the GLUT4 translocation measured in the current study appears to mirror the peak concentrations of plasma insulin, which occurred 30 min following glucose ingestion. In contrast to previous assumptions the results from this study suggest that GLUT4 translocation to the PM in response to physiological rises in plasma insulin following glucose feeding is transient and occurs following a similar temporal pattern to the peak plasma insulin concentration.

### Importance of peripheral GLUT4 localization

In previous rat and mouse studies, skeletal muscle GLUT4 was located predominantly at the fiber periphery in both the basal and stimulated states (Ploug et al. 1998; Lauritzen et al. 2006), but in these studies no evidence was presented that GLUT4 actually colocalized with a PM marker protein. The images presented in our study confirm that GLUT4 is also predominantly peripheral in human skeletal muscle fibers and in addition provide evidence that GLUT4 colocalizes with the PM marker dystrophin in both the basal and stimulated states. Two previous studies using different mouse models with specific GLUT4 exofacial labeling techniques have visualized the incorporation of GLUT4 in the PM and both detected GLUT4 in the PM in the basal state (Schertzer et al. 2009; Lizunov et al. 2012). This suggests GSV fusion and GLUT4 incorporation into the PM occur even in the basal state and are in agreement with data indicating a role for GLUT4 in basal glucose uptake (Hansen et al. 1995).

Large clusters of GLUT4 (>1 *μ*m in diameter) have been previously characterized as being present in membranes of the trans-Golgi network with small clusters (<1 *μ*m in diameter) identified as endosomal stores or GSVs (Ploug et al. 1998; Lauritzen et al. 2008). In the current study the number and size of large GLUT4 spots and size of small GLUT4 spots in the PM layer and five intracellular 1 *μ*m layers beneath the PM showed a reduction following 30 min exercise. This is consistent with previous reports in rodent muscle that exercise recruits GLUT4 from large and small clusters (Ploug et al. 1998). However, Ploug et al. (1998) also reported recruitment of GLUT4 from large and small clusters following insulin stimulation by means of a large bolus intravenous insulin injection (Ploug et al. 1998), which is in contrast to our study in which oral glucose ingestion leading to a modest increase in plasma insulin (Fig.3B) did not deplete the number or size of large or small GLUT4 spots.

In adipocytes a mode of GLUT4 exocytosis has been proposed to occur whereby in the basal state GLUT4 is retained in clusters in PM regions, while insulin stimulation increased the prevalence of these clusters but also elicited dispersal of GLUT4 molecules into the PM (Stenkula et al. 2010). The observations presented in the current study in human skeletal muscle have similarities to these data. Clusters of GLUT4 are clearly observed in PM regions in the basal state (Figs.1A,B and 4A,B). Thirty and 60 min following glucose ingestion, there is a significant increase in the number of large GLUT4 spots in the PM layer. In addition there is the appearance of continuous GLUT4 staining along the PM and a significant increase in the Pearson's correlation coefficient 30 min following glucose ingestion (Fig.4E,F). In contrast, following exercise the clusters of GLUT4 staining at the PM are absent and there is instead continuous homogenous GLUT4 staining along the PM, indicative of depletion of GLUT4 clusters and GLUT4 dispersal in the PM (Fig.1E,F). We suggest that the magnitude of GLUT4 translocation in human skeletal muscle fibers is greater following 30 min moderate intensity exercise than 30 min following glucose ingestion. The first line of evidence to support this is the reduction in large spot number, large spot size, and small spot size in all layers measured following 30 min moderate exercise. In comparison there is an increase in large spot number in the PM layer 30 min following glucose ingestion. Secondly, there is a significant difference between the magnitude of the increase in the Pearson's correlation coefficient following 30-min moderate intensity exercise and 30 min following glucose ingestion (mean increase following exercise *r = *0.11 ± 0.02, mean increase following glucose ingestion *r = *0.04 ± 0.01, independent samples *t* test *P < 0.01*). We propose that following moderate exercise, GLUT4 clusters fuse with the PM and most of the GLUT4 clusters are depleted, resulting in stronger dispersal of GLUT4 in the PM. In contrast, while stimulation with physiological increases in plasma insulin 30 min following glucose feeding recruits GLUT4 clusters to the PM, GLUT4 clusters are depleted to a lesser extent resulting in a weaker GLUT4 PM signal.

The importance of peripheral GLUT4 localization is highlighted by recent studies using TIRF microscopy. Lizunov et al. (2012) demonstrated that in skeletal muscle 80% of insulin-mediated GSV fusion events emanated from vesicles that were within 100 nm of the PM and pretethered at the PM prior to insulin stimulation (Lizunov et al. 2012). This explains why we observed an abundance of GLUT4 clusters in peripheral regions of unstimulated muscle fibers. It is important to note that the resolution limits of confocal microscopy (~200 nm) dictate that it is not possible to distinguish between a vesicle that is fused at the PM, pretethered at the PM or simply in close proximity to the PM (Schertzer et al. 2009). This caveat is particularly applicable to the basal state where GLUT4 is located in clusters that colocalize with dystrophin staining. In contrast, we propose that the appearance of continuous and homogeneous GLUT4 and dystrophin colocalization, only following glucose ingestion and exercise, is evidence of GLUT4 fusion and dispersal in the PM and overall net GLUT4 translocation.

## Conclusions

This study has, for the first time, successfully used an immunofluorescence technique to visualize GLUT4 translocation to the PM of human skeletal muscle measured as increases in GLUT4 and dystrophin colocalization using the Pearson's correlation coefficient 30 min following glucose ingestion and immediately after 30 min of moderate intensity exercise. In addition GLUT4 large spot number and size and small spot size was reduced in the intracellular layers immediately adjacent to the PM following 30 min moderate intensity exercise, but not 30 min after glucose ingestion. Together these observations are indicative of GLUT4 translocation mediated by insulin and muscle contraction. This methodology could permit future studies to investigate the relative contributions made by the various mechanisms that regulate glucose uptake in response to insulin and contraction in human skeletal muscle and the mechanisms that underlie the development of insulin resistance in obesity and aging.

## Conflict of Interest

None declared.
